# Case of Hepatic Disease

**Published:** 1864-10

**Authors:** D. B. Trimble

**Affiliations:** Chicago, Ill.


### ARTICLE XXXV.

### CASE OF HEPATIC DISEASE.

By D. B. TRIMBLE, M.D., Chicago, Ill.

Editor Medical Examiner:

Dear Sir:—If you think the following case, from my note book, will be of sufficient interest for insertion in your journal, it is at your service.

It is a case shewing the beneficial effects of free ptyalism in severe hepatic and gastric derangement; and of the recuperative powers of cod liver oil. In addition to its valuable influence in pulmonary affections, the excellent tonic and restorative qualities of the oil in cases of emaciation and debility from other causes, I have witnessed in a number of instances, of which the following case is one:—

I was sent for on the evening of April 3, 1859, to see a person, some miles from my residence, who was said to be "dying." I found the patient, Mrs. R., aged about thirty years, affected as follows: Intense pain and tenderness in the abdomen, particularly in the epigastrium; a circumscribed swelling in the upper part of the right iliac region; obstinate constipation; a weak, fluttering, and frequent pulse; tongue dry, and coated with a dark-brown fur; skin, and albuginea oculi, of a deep icterode hue; emaciation equal to that of a person in the last stage of phthisis; complete anorexia; and suppression of catamenia since preceding May.

The history of her case, as given me by herself, and afterwards confirmed by a conversation I had with her first physician, a gentleman of intelligence and experience, was, that she was taken sick early in May, 1858, and on the 25th of that month had a severe chill, when she first sent for her physician. The ague continued for a "considerable" time; she continued to grow worse, and suffered great pain in the epigastric and right hypochondriac regions, increasing paroxysmally, but never entirely intermitting. Her disease progressed; she became very prostrate, and greatly emaciated, and a consultation with an eminent practitioner was held, who concurred in the view of the attending physician, that it was a severe case of biliary calculi. The measures resorted to, partially and temporarily relieved the sufferings of the patient; but the emaciation continued and increased, and after a time the symptoms were renewed with greater severity. Her physicians then said they could do no more for her, and her husband called in another. The last practitioner attended her a few times, without apparent benefit, and then discontinued his visits; several weeks elapsed when they sent for me under the impression that she was dying.

After a thorough examination, I supposed it to be a case of chronic inflammation and induration of the liver, in which the stomach and bowels had become implicated. The severe paroxysms of pain, I supposed, might (as decided by the two gentlemen who first attended her,) arise from the passage of biliary calculi through the cystic duct, and ductus communis choledochus; and the irritation thus produced, combined with malarial influence, was no doubt the cause of her disease.

The immediate indications were, to allay the suffering, support the strength, and procure sleep; and I therefore prescribed mild stimulants and anodynes, Dovers powder, with wine-whey, and light liquid nourishment was my first prescription. The next day I found she had had a better night than usual; and I then directed 12 grains pill. hydrarg, to be followed by ol. ricini in five hours. As the case was almost a hopeless one, and believing that the liver must be brought into greater and healthier action, and the attending irritation of the stomach and bowels, be relieved to give any chance for a cure. I determined to push the mercurial treatment to ptyalism; supporting the strength at the same time, by as much nutriment of a proper character as the patient would bear. I therefore continued the pill. hydrarg., alternating with p. ipec. et opii., and applying ung. hydrarg. to right hypochondrium. In connection with this course I also administered taraxacum.

About this time the patient had a convulsion, and remained insensible for 18 or 20 hours. I did not see her while in this condition; her friends supposed her to be dying, and did not send for me. In a few days the breath and gums began to give evidence of the mercurial influence, and twenty-four hours after these symptoms appeared, I suspended the medicines, except the taraxacum. The salivation increased, and became severe; gave her beef tea, wine-whey, &c.; astringent gargles, and small doses of sol. sulph. morphia, for a few days to allay the abdominal pain, which had, however, greatly abated on the appearance of ptyalism. The bowels were now moved copiously, two or three times a day, without other medicine than a dose of castor oil once in two or three days. The fæces were dark, and of a tar-like consistence, gradually becoming more natural in appearance; the jaundiced color of the skin and eyes abated; the eyes had a more animated appearance, and the appetite rather better. The pulse became slower, and more regular, and the pain had nearly subsided.

I now commenced giving her half an ounce cod liver oil, with comp. tinc. cinch. twice a day; and she soon evinced the benefit she was deriving from it. The jaundiced hue of the skin and eyes disappeared; her appetite became urgent, so that it was necessary to restrain it; bowels were regular; pain relieved; her flesh and strength rapidly increased, and on the 20th May, I ceased attending her, with directions that the oil should be continued two weeks longer. I called to see her about the middle of June, but she was not at home. Her mother said "she was well, but swollen in the abdomen." On the 19th of July, I called again, and found her at her work, perfectly restored to health, and with quite sufficient flesh. Her catamenia had returned the week previous, the first time for 18 months, and the abdominal tumefaction had subsided.

I think the effects of the treatment in this case so evident, that there is little necessity for comment. That the salivation relieved the engorged and irritated condition of the liver, and the torpid state of the alimentary canal, there can be no doubt; and the system being thus relieved, the cod liver oil aided materially in her rapid restoration to health.

*Chicago, August* 17, 1864.

